# Analysis of Oral Leukoplakia and Tobacco-Related Habits in Population of Chengalpattu District- An Institution-Based Retrospective Study

**DOI:** 10.7759/cureus.25936

**Published:** 2022-06-14

**Authors:** Aarthi Venkat, Sathya Kumar M, Aravindhan R, Magesh K T, Sivachandran A

**Affiliations:** 1 Dentistry, SRM Kattankulathur Dental College and Hospital, Chennai, IND; 2 Oral Pathology and Microbiology, SRM Kattankulathur Dental College and Hospital, Chennai, IND

**Keywords:** prevalance, oral potentially malignant lesion, dysplasia, tobacco, oral leukoplakia

## Abstract

Objective

The study aimed to evaluate the prevalence of oral leukoplakia and to assess the risk of developing oral leukoplakia in patients with tobacco-related habits among the populations of Chengalpattu district, Tamil Nadu, India.

Materials and Methods

Incidence and prevalence of oral leukoplakia differ among different populations in India due to cultural and demographic diversities. The results obtained from this study can be used as a reference in future research and policy-making for tobacco control. Data for this study were manually ascertained from the Department of Oral Pathology and Microbiology, SRM Kattankulathur Dental College and Hospital, SRM Institute of Science and Technology, Potheri, Chengalpet district, Tamil Nadu, India. The medical records of the patients diagnosed with the abnormalities and diseases of the oral mucosa, especially the white lesions of the oral cavity, between January 2011- March 2021 (10 years). The basic inclusion criteria for this study were to include the histopathology reports of the white lesions with no malignant changes during clinical diagnosis. The data was analyzed based on age, gender, tobacco-related habits, and histopathological diagnosis. The exclusion criteria for the study were the cases reported as carcinoma, patients associated with syndromes, biopsies outside the mentioned period, and those patients with incomplete clinical or histopathological details.

Results

Among 141 white lesions, about 85 cases [60.2%] were confirmed as oral leukoplakia, of which the study population had 55 (64.7%) males and 30 (35.3%) females. The age group which was commonly seen was 41-60 years. About 80% of the population with oral leukoplakia had the habit of tobacco consumption. The use of tobacco products was seen more commonly in the male population than the female, and consumption of tobacco and alcohol was seen in 6% of the population. In our study, we found the study population had the habit of using smokeless tobacco rather than smoking cigarettes and bidis. About 20% of the population diagnosed with leukoplakia did not have any habits. The most commonly affected site was buccal mucosa (67%), followed by the tongue (12%).

Conclusion

Our study shows a statistical association between oral leukoplakia and tobacco product consumption among the population of Chengalpattu district. The oral health care providers must take utmost care and vigilance to diagnose the lesion at its earliest stage and give appropriate treatment modalities and effective tobacco interventions.

## Introduction

World Health Organization (WHO) defined precancerous lesion as "a morphologically altered tissue in which oral cancer is more likely to occur than its normal counterpart" and the precancerous condition as "a generalized state associated with significantly increased risk of cancer" [1]. In 2005, WHO recommended "Oral potentially malignant disorder" (OPMD) to avoid confusion between the terminologies. Oral leukoplakia is the commonest OPMD [2]. In 1861, Karl Freiherr von Rokitansky used the term leukoplakia to refer to the white lesions of the urinary tract. Later, Schwimmer, in 1877, was the first person to use the term for an oral white lesion [3]. In 1994, WHO defined leukoplakia as "a predominantly white lesion of oral mucosa that cannot be characterized as any other definable lesion clinically or pathologically, often associated with tobacco products, some of which will transform into cancer".Tobacco was introduced in Europe in the late fifteenth century and was later introduced to the Indian market by Portuguese traders within the late sixteenth or early seventeenth century. Tobacco usage is significant in India as it is the second-largest producer after China [4].

The global adult tobacco survey (GATS) by the Ministry of Health and Family Welfare, Government of India, found that the prevalence of tobacco use among Indian adults aged above 15 years was 35% in 2010 and 29% in 2017. Khaini is the most commonly used tobacco product, followed by bidi smoking and gutka among Indian men. The use of smokeless tobacco is more common among Indian men and women than smoking types [5].

Other well-known etiological features of oral leukoplakia are alcohol, microbial infection, chronic irritability (improper dentures), malnutrition, UV radiation, and galvanism [6]. Based on the clinical features, oral leukoplakia can be divided into a flat and uniform white known to be homogenous and non-homogeneous type appears as a white and red lesion ("erythroleukoplakia") which may either be speckled or nodular. Leukoplakia is a clinical term. A histopathological examination of the tissue must be performed to ascertain the exact nature of the lesion. Although epithelial dysplasia may also be reported, it is not seen in all cases of oral leukoplakia. The presence of these dysplastic changes reveals the disturbance in the homeostatic control mechanism of the epithelium and is an important predictor of malignant development in the premalignant lesion.

Although OPMDs and cancer are common in India, the epidemiological data reported in the literature may not be the same among various geographical areas. The present study attempts to update the prevalence of oral leukoplakia in the people residing in the Chengalpattu district, Tamil Nādu, and to analyze the usage of tobacco-related products in this population.

## Materials and methods

Data for this study were manually ascertained from the Department of Oral Pathology and Microbiology, SRM Kattankulathur Dental College and Hospital, SRM Institute of Science and Technology, Chengalpattu district, Tamil Nādu, India. As the retrospective study does not involve human samples or tissues, the Institutional review board's clearance was not required. The data were analyzed regarding age distribution, gender, personal habits, and histopathology grades. The inclusion criteria included all the clinically visible white oral cavity lesions provisionally diagnosed as oral leukoplakia and confirmed by histopathological examination. The exclusion criteria for the study were the cases reported as carcinoma, patients associated with syndromes, biopsies outside the mentioned period, and those patients with incomplete clinical or histopathological details. The histopathological grading was listed as hyperplasia, mild dysplasia, moderate dysplasia, and severe dysplasia.

Statistical methods

Qualitative and quantitative variables did the statistical analysis. Both Pearson's and spearman's correlation can be used to determine the strength of the two variables. In our study, we used Pearson's correlation coefficient, it was used to determine the strength and relationships between variables. Each characteristic or variable (age, site, etc.) was analyzed with others to find any significant association among them. The statistical analysis between two variables was calculated using the chi-square test, and the Chi-square test was done between the duration of habits and histopathological diagnosis, p < 0.05 was considered the statistical significance level in all the calculations. 

## Results

A total of 2376 oral biopsies were reported from January 2011 to March 2021. Of that, 141 were clinically diagnosed as white lesions, including oral leukoplakia, lichen planus, oral submucous fibrosis, and tobacco pouch keratosis. Among 141 white lesions, about 85 cases, that is, 60.2% were diagnosed as oral leukoplakia, followed by oral lichen planus (20%), and oral submucous fibrosis (14.8%), and tobacco pouch keratosis (5%). The overall prevalence of oral leukoplakia was found to be 3.57% in our study group (Table 1).

**Table 1:** Year-wise distribution of patients reported with Oral Leukoplakia

Year	Patients with oral leukoplakia	Male	Female	
Mar-21	3	2	1	
2020	5	3	2	
2019	14	9	5	
2018	5	4	1	
2017	7	5	2	
2016	11	3	8	
2015	11	7	4	
2014	13	12	1	
2013	10	7	3	
2012	4	2	2	
2011	2	1	1	
Total	85	55	30	

In our study, Oral leukoplakia was most commonly seen in men (64.7%) than women (35.3%). A higher male predilection was noticed among the middle-aged groups; 77.7% of the study participants were within 21- 40 years, and 65.9% in 41-60 years. In the last decade, no cases were reported among young adolescents (<20 years). In contrast, slight female predilection (58.8%) was noted among elderly individuals (>60 years). Around 48.2% of cases were reported among the 41-60 age group, which was found to be the most common age group affected with oral leukoplakia in our study (Table 2).

**Table 2:** Age and Gender wise distribution of patients with Oral Leukoplakia

Age Group (in Years)	Male	Female	Total
21-40	21 (77.7%)	6 (22.2%)	27
41-60	27 (65.85%)	14 (34.1%)	41
>60	7 (41.1%)	10 (58.8%)	17
Total	55 (64.7%)	30 (35.3%)	85 (100%)

**Figure 1:**
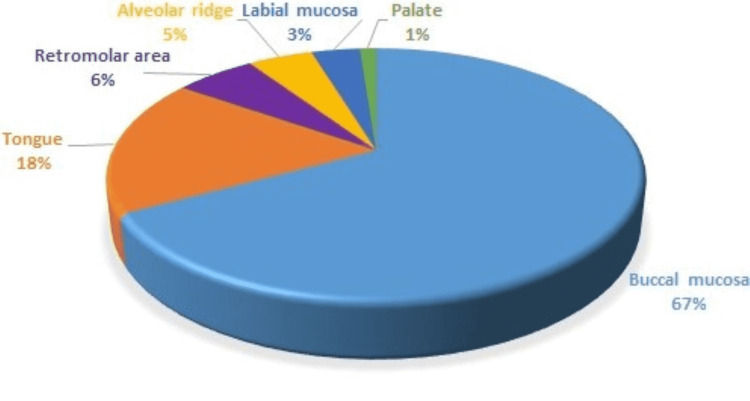
Site-wise distribution of Oral Leukoplakia

Around 80% of the study population with oral leukoplakia were associated with some form of tobacco consumption, including cigarette, bidi smoking, pan, and tobacco chewing habits. Males show a significant surge (94.5%) in the usage of tobacco products than females (53.3%). Smokeless tobacco consumption was more commonly used by the study participants (53%) than smoking habits (47%). Sharp teeth, ill-fitting dentures, and idiopathic factors were some of the commonest etiological factors for patients with lesions but did not have any associated habits (20%). The duration of the associated habits ranges from 2 years to 15 years, with the majority having a slower lesion evolution. The commonest site being involved with the leukoplakia was the buccal mucosa (67.05%), followed by the tongue (17.6%), retromolar area (5.8%), alveolar ridge (4.7%), labial mucosa (3.5%) and palate being the least affected site (1.1%) in our study (Figure 1).

On histopathological analysis, about 41% of the population showed hyperkeratotic changes in the affected area, and an equal percentage reported mild dysplastic changes. About 14% of the population had moderate dysplasia, and about 4% of the population showed severe dysplastic features in our study.

## Discussion

On histopathological analysis, about 41% of the population showed hyperkeratotic changes in the affected area, and an equal percentage reported mild dysplastic changes. 14% of the population had moderate dysplasia, and about 4% of the population showed severe dysplastic features in our study.

The term "Leukoplakia" is derived from Greek literature 'Leucos' means white and 'Plakia' means patch. Leukoplakia is one of the well-documented oral potentially malignant disorders (OPMD) globally and is more prevalent in India due to its cultural, ethnic, and geographic factors [7]. The causes of oral leukoplakia are multifactorial such as tobacco consumption, ill-fitting dentures, microbial agents, genetic causes, and usage of sanguinaria [6].

Recent studies showed that about 4.47% of patients globally have OPMD, predominantly among men and the Asian population [8]. Only around 5% of oral pathoses constitute white lesions, among which leukoplakia, verrucous leukoplakia, and lichen planus have malignant potential as high as 0.5-100%. Therefore, the dentist plays a crucial role in diagnosing oral lesions to eliminate the risk of malignancy as soon as possible. The present study shows about 85 cases (3.57%) of the total 2376 patient data analyzed with oral leukoplakia (Table 1).

In the systematic review, Petti et al. [9] stated that the frequency of oral leukoplakia was between 1.7% and 2.7% globally. Several Indian studies have shown the prevalence of leukoplakia ranges between 0.21% and 5.22%. In our study, we could see that males were more commonly affected (64.7%) than their counterparts. We could also notice that leukoplakia significantly occurred in the age group of 41-60 (48%). Mello et al. [10] concluded in their systematic review that oral leukoplakia and other potentially malignant oral diseases often affect patients over 50 [2].

Wang et al. [11] found that women were more commonly affected than men and also noticed that the malignant transformation rate remains high among females. In our retrospective study, we found that the incidence of oral leukoplakia remains high in males among middle-aged adults (between 21-40 and 41-60). In contrast, slight female predilection was noted only in older individuals (>60 years).

In their study, Rubert et al. [12] mentioned that gender may play a role in the risk of malignant transformation. However, in contrast, the studies by Jayasooriya et al. [13] and Matulic et al. [14] stated that gender is not related to malignant transformation. A higher male predilection was noticed among the middle-aged groups; 77.7% of the study participants were within 21- 40 years, and 65.9% in 41-60 years. Also observed in our study, in the last decade, no cases were reported among young adolescents (<20 years) (Table 2).

Oral leukoplakia is usually found locally or spread across the buccal mucosa, lip commissures, or gingiva. The commonest involved site was the buccal mucosa (67.05%), followed by a tongue (17.6%), retromolar area (5.8%), alveolar ridge (4.7%), labial mucosa (3.5%), and palate being the least affected site (1.1%) in our study (Figure 1 ). Similar observations were noted in those studies conducted by Ramya et al. [15], Gopinath et al. [16], and Sivakumar et al. [17]. 

Literature shows that around 35% of Indian adults were using tobacco [WHO]. In our study, we found that around 80% of the study population with oral leukoplakia were associated with some form of tobacco consumption, including cigarette, bidi smoking, pan, and tobacco chewing habits. Males show a significant surge (94.5%) in the usage of tobacco products than females (53.3%). Smokeless tobacco consumption was more commonly used by the study participants (53%) than smoking habits (47%). The study by Mini et al.,2014 [18] and Sujatha et al.,2012 [19] showed that, unlike western countries, in Asian countries, smokeless tobacco such as areca nut and betel quid is used predominantly [1]. Literature shows that most patients who presented with leukoplakia had a smoking history. A pattern of tobacco usage varies significantly in India based on social, cultural, economic, and geographical distribution.

According to Petti et al. [9], the severity of the oral lesions listed as OPMD subsides on eliminating the risk factors such as the use of tobacco, consumption of alcohol, betel nut, and combination of the habits. Dogenski et al. 2020 [3], in their study, recorded that tobacco usage contributed about 44.70% to causing oral leukoplakia, followed by idiopathological factors (28.03%), alcohol consumption (9.85%), and sun exposure (9.09%). 

There is no association between the location of the lesion and dysplastic changes. The lesion's clinical forms, such as thickness, granularity, and those admixed with red spots (erythematous area), show dysplastic changes compared to homogenous forms [20]. However, Rubert et al. 2020 [12] conducted a retrospective study on a group of 412 patients who were diagnosed with oral leukoplakia, where about 53.2% were nonsmokers [2]. In our study, about 20% of patients diagnosed with leukoplakia did not reveal any associated habits.

In our study, we found Homogenous type of leukoplakia predominates over non-homogenous forms; also, we observed the duration of the associated habits ranges from 2 years to 15 years, the majority having a slower evolution of the lesion. The rate of malignant transformation of leukoplakia ranges from 0.12% to 17.5% among the studies reported in the literature [21]. A meta-analysis done by Pinto et al. 2020 [22] revealed that statistically, there was no signiﬁcant association between the gender and frequency of malignant transformation of oral leukoplakia; however, the odds ratio favored males, which estimated around 9.7% [2].

Nutritional compromise and psychologic stress complicate the impact and frequency of the habits and make leukoplakia an early harbinger of oral malignancy [23]. Histological examination using biopsied tissue is the gold standard confirmatory procedure for diagnosing oral lesions. On histopathological analysis, about 41% of the population showed hyperkeratotic changes in the affected area, and an equal percentage reported mild dysplastic changes. 14% of the population had moderate dysplasia, and about 4% of the population showed severe dysplastic features in our study. To date, it is unclear which and when these OPMD lesions turn into malignancy. 

Even though there were many ways of treating oral leukoplakia, relapses and adverse effects were common. Henceforth Policy makers and Healthcare providers should work towards quitting and avoidance of tobacco usage. Patients who cannot improve their oral health and lifestyle are advised of rehabilitation programs. It is essential to educate the general public, the Young population, and the school-going children regarding the ill effects of using tobacco-related products, which will significantly reduce tobacco usage.

Limitations

The limitation of this study includes the unavailability of information on other associated risk factors such as oral hygiene practices, mechanical trauma, and synergistic effects of alcohol consumption. As it was a retrospective study, data taken in the study was from a single institution, and it did not reveal the true prevalence among the general public.

## Conclusions

This retrospective, Institution-based study highlights the increased association of tobacco-related products among the patients reported with oral leukoplakia. Despite the measures taken by the Government of India to control tobacco usage, India remains the second-largest tobacco consumer in the world. Also, the awareness regarding the ill effects of tobacco among the general public remains low. Dentists play a major role in controlling and preventing tobacco-induced lesions through patient education, counseling, cessation assistance, mass screening, community-based educational programs, early detection of the lesions, and appropriate treatment procedures.
